# Population genetics of *Anopheles arabiensis*, the primary malaria vector in the Republic of Sudan

**DOI:** 10.1186/s12936-021-03994-7

**Published:** 2021-12-19

**Authors:** Mashair Sir El Khatim Mustafa, Zairi Jaal, Sumia Abu Kashawa, Siti Azizah Mohd Nor

**Affiliations:** 1 Department of Biology & Chemistry, Al Butana University, 200, Rufaa, Sudan; 2 School of Biological Sciences, Universiti Sains Malaysia, 11800 Penang, Malaysia; 3 Faculty of Science, Department of Zoology, University of Khartoum, Khartoum, Sudan; 4 Institute of Marine Biotechnology, Universiti Malaysia Terengganu, 21030 Kuala Terengganu, Malaysia

**Keywords:** *Anopheles arabiensis*, Microsatellite loci, Population genetic, Gene flow, Bottleneck, Sudan

## Abstract

**Background:**

*Anopheles arabiensis* is a member of *Anopheles gambiae* complex and the main malaria vector in Sudan. There is insufficient population genetics data available on *An. arabiensis* for an understanding of vector population structure and genetics, which are important for the malaria vector control programmes in this country. The objective of this investigation is to study the population structure, gene flow and isolation by distance among *An. arabiensis* populations for developing control strategies.

**Methods:**

Mosquitoes were collected from six sites located in three different states in Sudan, Khartoum, Kassala and Sennar, using pyrethrum spray catch of indoor resting mosquitoes. Anopheline mosquitoes were identified morphologically and based on species specific nucleotide sequences in the ribosomal DNA intergenic spacers (IGS). Seven published *An. gambiae* microsatellite loci primers were used to amplify the DNA of *An. arabiensis* samples.

**Results:**

PCR confirmed that *An. arabiensis* was the main malaria vector found in the six localities. Of the seven microsatellite loci utilized, six were found to be highly polymorphic across populations, with high allelic richness and heterozygosity with the remaining one being monomorphic. Deviation from Hardy–Weinberg expectations were found in 21 out of 42 tests in the six populations due to heterozygote deficiency. Bayesian clustering analysis revealed two gene pools, grouping samples into two population clusters; one includes four and the other includes two populations. The clusters were not grouped according to the three states but were instead an admixture. The genetic distances between pairs of populations ranged from 0.06 to 0.24. Significant F_ST_ was observed between all pairwise analyses of *An. arabiensis* populations. The Kassala state population indicated high genetic differentiation (F_ST_ ranged from 0.17 to 0.24) from other populations, including one which is also located in the same state. High gene flow (Nm = 1.6–8.2) was detected among populations within respective clusters but limited between clusters particularly with respect to Kassala state. There was evidence of a bottleneck event in one of the populations (Al Haj Yousif site). No isolation by distance pattern was detected among populations.

**Conclusions:**

This study revealed low levels of population differentiation with high gene flow among the *An. arabiensis* populations investigated in Sudan, with the exception of Kassala state.

## Background

In Sudan, malaria remains as one of the most important public health problems. More than 400 species of *Anopheles* have been recognized globally and, of these, 41 species can transmit malaria but only a few of them are malaria vectors [1]. With the exception of Southern Sudan, the main malaria vector in Sudan is *Anopheles arabiensis* [2–9]. However, in Southern Sudan *Anopheles gambiae* and *Anopheles funestus* are the major vectors of malaria and their vectorial capacity may be similar to that of *An. arabiensis* [1]*.* This species is a highly adaptable species with the capability to feed on multiple host species, both indoors and outdoors and acclimatize to a wide range of larval habitats [10, 11]. At present, indoor residual spraying (IRS) and insecticide treated bed nets (ITNs) are the main methods extensively used for vector control worldwide. These methods have proven valuable in reducing malaria burden [12, 13], but their usefulness has been threatened by increasing prevalence of insecticide resistance in the most important malaria vectors, including *An. arabiensis*. Thus, there is an urgent need for effective and sustainable alternatives to these traditional vector control strategies.

Microsatellites are genetic markers of short tracts of tandemly repeated DNA sequences. These markers have become the genetic marker of choice for studying the population genetics of many eukaryotic species, including mosquitoes. For instance, they have been widely utilized in such studies in the *An. gambiae* complex [14–20]. They have been developed into PCR-based molecular markers that are very useful for small organisms with limited extractable DNA [21].

To date, 150 polymorphic microsatellite loci have been characterized in *An. gambiae *sensu lato (s.l.) [14, 22], which have been widely used to explain the population structure and gene flow within and between members of the *An. gambiae* complex [23–25]. Most of these studies have been conducted on *An. gambiae* with some limited data on *An. arabiensis*. Present literature on *An. arabiensis* has revealed the lack of subpopulation differentiation in relation to larval habitat utilization [24]. Lack of annual bottlenecks in response to changes in the environment has also been documented [17, 26]. Large effective population size and/or recent range expansion as opposed to group migration [26, 27] have been attributed to the lack of population structuring. This is based on several experimental studies which have reported a short flight range for this malaria vector species [28] among villages in The Gambia. On the other hand, there is evidence in support of population structuring [29] from West Africa and eastern outer islands [27] of Eastern Africa. Furthermore, limited gene flow has been observed between the west and south east of the Rift Valley and in Southern Zambia [17, 23], respectively. Geographic distance and habitat alterations have been suggested as the main contributors of genetic isolation.

*Anopheles arabiensis* has changeable deme sizes ranging from as low as 25 km [27] to a few 1000 kms [17]. It was observed that in the Mwea Rice Scheme of Central Kenya, *An. arabiensis* mosquito densities decrease with increasing distance from the scheme [30, 31]. On the contrary, the human blood index [10] in addition to malarial transmission [30] by this species were significantly lower inside than in the outer areas of the rice scheme. All these factors influence mosquito reproductive fitness, survivorship and fertility [32]. Such alteration may change malarial transmission indices [33] and can lead to subpopulation differentiation [34–36] as was observed in this agricultural scheme. Moreover, the lack of evident geographical barriers that could have restricted gene flow between mosquito populations in the surrounding areas had led to the generation of a single panmictic population. A number of studies, for example one that was conducted by Dolo et al. [34] in the irrigated area of Sahel in Mali had suggested that the existence of mosquito colonies in an adjoining non-irrigated area during the dry season was maintained through migration of a few individuals from the irrigated areas.

Several comparative population studies between *An. arabiensis* and *An. gambiae* have shown a higher level of genetic differentiation in the latter species. Significant genetic differentiation, F_ST_ = 0.072–0.100 were observed for *An. gambiae* populations between western Kenya and coastal Kenya using microsatellite markers. Lehmann et al. [35, 36] suggested the Great Rift Valley as a major gene flow barrier for this species. However, non-significant genetic differentiation was identified for *An. arabiensis* populations from the two areas using the same loci [23]. Similarly, Donnelly and Townson [25] noted non-significant genetic differentiation of *An. arabiensis* populations in Malawi and Sudan. It thus appeared that different mechanisms of gene movement were in operation between the two species. Considering *An. gambiae*, these studies were discordant with another study on *An. gambiae *sensu stricto (s.s.) (mean F_ST_ = 0.006), which was genetically non differentiated across the 6650 km^2^ of the Kilombero valley landscape southern Tanzania. This suggested that the genetic differentiation in other populations was not due to physical barriers or distance. One plausible explanation is that there was environmental diversification even within the Kilombero valley [19]. Thus, the differentiated populations of *An. gambiae* could have been maintained by some degree of reproductive isolation.

With respect to *An. arabiensis,* several studies have reported varying levels of genetic differentiation. Nyanjom et al. [13] detected low F_ST_ but statistically significant genetic structure for *An. arabiensis* populations in Ethiopia and Eritrea. On the other hand, Simard et al. [24] reported high levels of genetic differentiation in two island populations of *An. arabiensis* populations that were 240 km apart in the Indian Ocean (F_ST_ 0.080–0.215). High levels of genetic differentiation were also detected among *An. arabiensis* populations (mean F_ST_ = 0.066) in Kilombero valley southern Tanzania [19].

Therefore, the objective of this investigation was to study the population structure and gene flow among *An. arabiensis* populations in Sudan based on microsatellite markers which may assist in developing control strategies.

## Methods

### Study areas

**Fig. 1:**
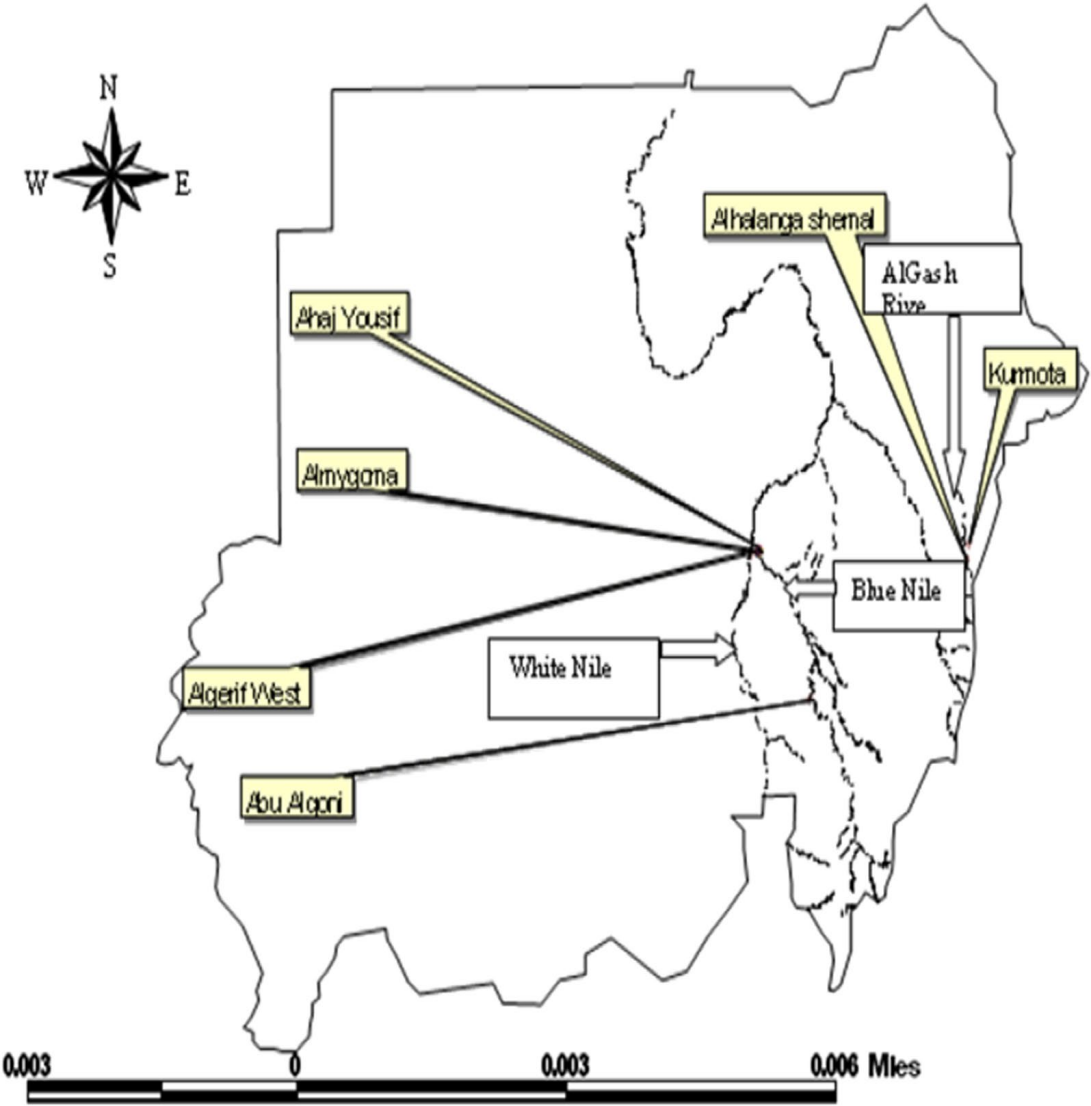
Study areas

A total of 200 specimens of *An. arabiensis* were collected from June 2010 to May 2011 from six different localities in Sudan representing different ecological zones separated by the River Nile and its tributaries (Fig. 1). Three localities were located in Khartoum State: (1) Mygoma (My), (2) Al Haj Yousif (Hj), and (3) El Gerif West (Gw). My and Hj are nearest to each other and located east of the Blue Nile. Al Haj Yousif (Hj) is northeast of Helt Koko, where animals are bred for milk production in a rich green area on the west bank of the Blue Nile. Two localites were located in Kassala State: (4) Alhalang Shemal (H.sh), an area of non-agricultural land on the east bank of the Al Gash River and (5) Alkrmota (Kr) on the west bank of the Al Gash River in the centre of an agricultural area which is surrounded by groves of fruit and vegetables. The sixth population was located in the Sennar state (Se): (6) Abu Algoni (Se), a farming area on the west bank of the Blue Nile River. Khartoum is the most central among the three regions while Kassala is in eastern Sudan with Sennar lying between the two, further south. The Blue Nile flows along Khartoum and Sennar while its tributary the Al Gash River flows along Kassala. The pairwise geographical distances between the six localities ranged from 3.93 to 569.25 km.

### Microsatellite PCR amplification

Molecular classification of *An. gambiae* species complex in this study was conducted based on the ribosomal DNA intergenic spacers (IGS) [37]. DNA extraction from individual *Anopheles* was conducted using the DNeasy blood and Tissue E kit (QIAGEN, Valencia, CA). Seven published *An. gambiae* microsatellite loci primers [12] were used to amplify the DNA samples. The PCR reactions were performed in a gradient thermal cycler (MJ Research PTC-200 Peltier Thermal Cycler) for 30 cycles. The PCR mix contained 1 uL of genomic DNA, 5× PCR buffer (Promega, Madison, WI), 15 pmol of each fluorescent labelled (NED, HEX or FAM) forward primer, 200 mmol each dATP, dCTP, dGTP and dTTP, 1.2 uL of 25 mm MgCl_2_ and 0.5 U *Taq* DNA polymerase (Promega, Madison, WI) in a 20 uL total reaction volume. Singleplex PCR amplification was conducted for loci AGXH678, AG2H290, AG2H603, AG2H143, AG3H29, AG3H45, AG3H158 (Table 1). The thermal cycling conditions were; an initial hold at 95 °C for 2 min, followed by 30 cycles of 94 °C for 30 s, 55 °C for 30 s and 72 °C for 30 s and a final extension at 72 °C for 5 min. Satisfactory PCR products as detected in a 2% agarose gel were sent to the service provider (First BASE Laboratories Sdn. Bhd., Selangor, Malaysia) into two primer multiplex sets for fragment analysis. Set A contained a mixture of AGXH678, AG2H290 and AG3H45, while set B was a mixture of AG2H603, AG2H143, AG3H29 and AG3H158. Loci AGXH678, AG3H29, AG3H45 and AG3H158 are found outside the inversion regions of the chromosome, while AG2H603 and AG2H143 loci are found within fixed inversion of chromosome 2La, and AG2H 290 in the 2R polymorphic inversion.

**Table 1:** Allelic richness (A_R_), number of alleles (N_A_), and the mean allele number of observed (H_O_) and expected heterozygosity (H_E_) at each locus per population

Locus	Pop	Gw	H.sh	Hj	My	Se	Kr	Total
	N	33	34	32	34	33	34	200
AGXH678	A_R_	2	6	1	8	7	7	8
	N_A_	3	4	2	5	5	4	7
AG2H290	A_R_	1	3	2	3	3	3	3
	N_A_	2	4	3	4	4	4	4
AG2H603	A_R_	3	6	4	4	4	4	6
	N_A_	4	7	5	5	5	5	7
AG2H143	A_R_	6	12	6	10	12	12	15
	N_A_	4	5	4	4	7	7	13
AG3H29	A_R_	–	1	–	–	–	1	1
	N_A_	–	2	–	–	–	2	2
AG3H45	A_R_	10	10	4	9	10	10	13
	N_A_	6	5	3	7	6	5	13
AG3H158	A_R_	4	4	4	4	5	6	6
	N_A_	3	3	3	3	5	5	7
Mean	A_R_	4.3	6	3.5	6.3	6.8	6.1	7.4
	N_A_	3.7	4.3	3.3	4.7	5.3	4.6	7.6
	H_O_	0.55	0.55	0.6	0.63	0.67	0.63	0.61
	H_E_	0.57	0.55	0.56	0.62	0.59	0.61	0.58

### Data analysis

Allelic data scoring of alleles was carried out by inspection of the electrophoretograms as described in Arif et al. [38]. Two well-resolved peaks indicate a heterozygous individual while presence of a single main peak represent a homozygous individual. Minor bands or stutters are also often present but being non alleles, were not included in the input data. Screening of all the genotypic data was executed using Micro-Checker v2.2.3 [39] to check for presence of null alleles and stuttering or large allele dropouts. The Monte Carlo simulation method was applied to generate expected homozygote and heterozygote frequencies of alleles. The HWE analysis was used to assess deviation from expected allele frequencies and the frequency of any null allele detected with significance level at p < 0.05 obtained through 1000 permutations. To ensure compatibility with different software analyses the raw data was converted into several specific data formats using CONVERT [40]. Significant relationship between alleles at any two loci was tested using the likelihood ratio test of linkage disequilibrium based on Expectation–Maximization (EM) algorithm [41]. This was applied to all pairwise comparisons of loci using Arlequin version 3.11 [42] with 10,000 permutations followed by false discovery rate (FDR) adjustment [43] at 95% significant level.

Population genetic diversity was measured based on allelic richness (A_R_), adjusted for different sample size and number of alleles (N_A_). To test for global deviation from HWE in a population, the inbreeding coefficient, (F_IS_) for each locus and population [44] was estimated in FSTAT v.2.9.3 [45]. Mean genetic heterozygosity, observed (*H*_*O*_) and expected (*H*_*E*_) heterozygosities per locus and population were estimated over all loci. Testing of deviation from Hardy–Weinberg equilibrium (HWE) was conducted using the exact tests with 10,000 steps in Markov chain and 10,000 dememorization steps in Arlequin version 3.11 [42]. Multiple testing of HWE was adjusted using False discovery rate (FDR) corrections with a global significance level of 0.05.

Estimates of population differentiation, using Wrights F_ST_ [46] over all loci were conducted. F_ST_ is based on the infinite allele model (IAM) which hypothesizes that each new allele is generated at a given rate, µ [47].

The program BOTTLENECK V 1.2.02 [48] was used to detect whether the populations had experienced recent effective population size reduction. Two-phase (TPM) models, infinite allele (IAM) and stepwise mutation (SMM), and deviation from HWE was estimated using a two-tailed Wilcoxon sign-rank test followed by FDR adjustment. Qualitative descriptor of allele frequency (“mode-shift” indicator) was also performed in BOTTLENECK to discriminate “shifted mode” populations (bottleneck) from stable populations [48]. Mantel test was used to investigate the correlation between geographical and genetic distances among populations using Arlequin 3.11 [42].

Assignment of individuals to their respective source populations based on multilocus genotypic data was determined in STRUCTURE version 2.3 [49]. An assumption of correlated allele frequency among populations [49] and admixture model was used with the burn in period of 10,000 and MCMC length of 10 iterations. The probabilities of genotype assignment into each individual group were performed across replicates using CLUMPP version 1.1.2 [50] and the graphical presentation was carried out using Structure Harvester [51]. Ten independent runs were computed for all possible values of the maximum number of clusters (K = 6). Finally, based on genetic distance a neighbour-joining tree was constructed to determine the phylogenetic tree among the six populations using MEGA 5.0.5 [52].

## Results

All 200 individuals from six populations of *An. arabiensis* in Sudan were successfully genotyped and scored for all seven microsatellite loci. No evidence for scoring error due to null alleles, large allele dropout or stuttering was detected after assessing with Microchecker.

### Allelic frequency distribution and linkage disequilibrium

**Fig. 2:**
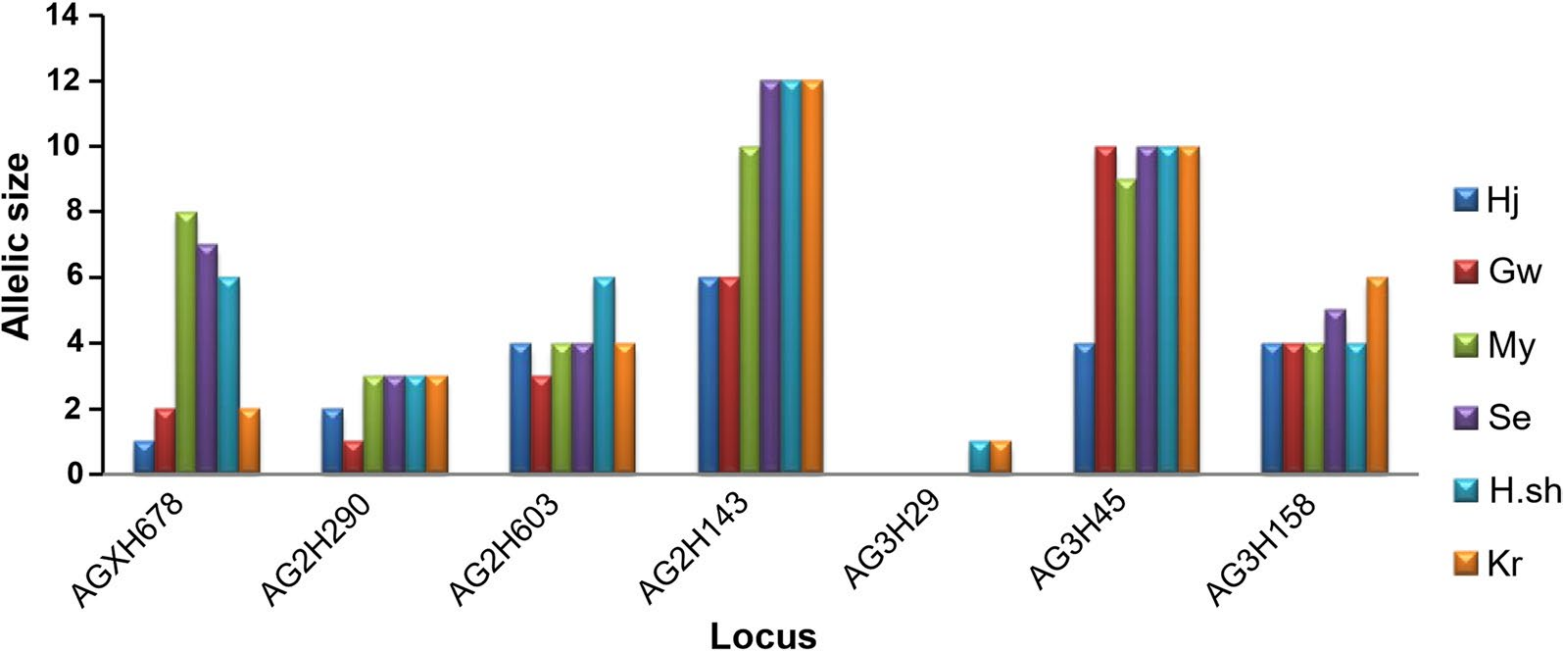
Number of alleles observed in each locus for each population

All (except one) microsatellite markers of *An. arabiensis* populations were found to be polymorphic in at least one population. However, locus AG3H29 was monomorphic in most populations. Loci AG2H143 and AG3H45 were moderately polymorphic with number of alleles per locus ranging from 6–12 and 4–10, respectively. The total number of alleles per locus ranged from 2 to 12 with an average of 7.6. The mean A_R_ ranged from a minimum of 3.5 in Hj (Khartoum State) to a maximum of 6.8 in Se (Sennar State) and the means observed heterozygosity of alleles per locus ranged from 0.55 to 0.67, while means of expected heterozygosity ranged from 0.55 to 0.62 (Table 1 and Fig. 2). Tests for linkage disequilibrium revealed that 14 pairwise comparisons (11.11% out of 126 pairwise comparisons) after Bonferroni correction were significantly deviated from the random association of alleles at two or more loci with the highest linkage disequilibrium detected from pairwise loci comparisons in Kr (5 pairs in loci AGXH678, AG2H290, AG2H143 and AG3H158), followed by My (4 in loci AGXH678, AG2H290, AG2H603 and AG2H143), H.sh (2 pairs in loci AG2H290, AG2H603), Hj (1 pair in AG3H158) and Se (2 pairs in loci AGXH678 and AG2H143). No linkage disequilibrium of loci was observed in Gw.

### Hardy–Weinberg equilibrium (HWE) and FIS

Each population was tested separately for significant departure from HWE at each locus. Observed heterozygosity varied from 0.12 to 0.94 while expected heterozygosity ranged from 0.11 to 0.76 (Table 2). Deviations from HWE were found in 21 out of 42 tests in the six populations. These were observed in locus AGXH678 (Gw and H.sh), locus AG2H290 (H.sh, Hj, My and Kr), locus AG2H603 (Gw, H.sh, Hj, My and Kr) locus AG2H143 (Gw, Hj, My and Se), locus AG3H45 (H.sh, Hj, My, Se and Kr) and locus AG3H158 in Kr. Thus, there was no consistent pattern according to locus or population and fairly equal numbers of heterozygote deficiencies and heterozygote excess were observed. Based on the Microchecker results, these deviations were presumably due to population subdivision rather than the existence of null alleles. Locus AG3H29 showed no deviations from Hardy–Weinberg equilibrium and this locus was monomorphic in all populations except in H.sh and Kr populations (both in Kassala State). Inbreeding coefficient (F_IS_) over all loci showed that most of the populations had high excess of heterozygosity (negative F_IS_ negative values indicate no inbreeding).

**Table 2:** Population genetic diversity as measured by observed (*H*_*O*_) and expected (*H*_*E*_) heterozigosities and F_IS_

	Pop	Gw	H.sh	Hj	My	Se	Kr
Locus	No	33	34	32	34	33	34
AGXH678	H_O_	0.49	0.85	0.44	0.63	0.58	0.64
	HE	0.53	0.65	0.48	0.63	0.60	0.53
	F_IS_	0.09	− 0.32	0.08	0.01	0.04	− 0.20
	P	0.03*	0.00*	0.71	0.18	0.16	0.19
AG2H290	HO	0.61	0.50	0.53	0.56	0.70	0.67
	HE	0.49	0.63	0.60	0.61	0.70	0.65
	F_IS_	− 0.26	0.21	0.12	0.08	0.01	− 0.03
	P	0.27	0.00*	0.03*	0.00*	0.10	0.00*
AG2H603	H_O_	0.61	0.65	0.69	0.84	0.79	0.75
	HE	0.70	0.68	0.73	0.76	0.72	0.77
	F_IS_	0.14	0.05	0.06	− 0.11	− 0.10	0.03
	P	0.00*	0.00*	0.00*	0.00*	0.12	0.00*
AG2H143	H_O_	0.58	0.62	0.88	0.67	0.55	0.58
	HE	0.65	0.68	0.61	0.51	0.50	0.63
	F_IS_	0.12	0.09	− 0.44	− 0.30	− 0.08	0.07
	P	0.001*	0.20	0.00*	0.00*	0.02*	0.38
AG3H29	H_O_	–	0.12	–	–	–	0.08
	HE	–	0.11	–	–	–	0.08
	F_IS_	–	− 0.05	–	–	–	− 0.03
	P	–	1	–	–	–	1
AG3H45	H_O_	0.52	0.53	0.75	0.69	0.58	0.94
	HE	0.50	0.63	0.55	0.74	0.70	0.76
	F_IS_	− 0.04	− 0.35	0.16	− 0.32	− 0.27	− 0.05
	P	0.73	0.00*	0.01*	0.00*	0.00*	0.00*
AG3H158	H_O_	0.49	0.59	0.31	0.66	0.58	0.72
	HE	0.53	0.44	0.37	0.50	0.46	0.69
	F_IS_	0.09	− 0.35	0.16	− 0.32	− 0.27	− 0.05
	P	0.22	0.09	0.07	0.09	0.20	0.00*

*H*_*O*_ = observed heterozygosity_,_ *H*_*E*_ = expected heterozygosity, F_IS_ = inbreeding coefficient, high positive values indicate deficiency of heterozygotes, while small or negative values indicate excess of heterozygotes

p = significance of deviation from HWE (p < 0.05), * = significant deviation from HWE

### Genetic structure

A hierarchical AMOVA performed after defining into three groups (Khartoum, Sennar and Kassala) revealed that 2.75% of the total genetic variance (F_CT_) was contributed by ‘among groups’ variation, 13.61% (F_SC_) was ‘among populations within group’ variation (Table 3) while 83.63% was attributed to ‘between individuals within population’ i.e. intrapopulation variation. All hierarchical levels i.e. F_SC_ and F_CT_ and ‘within population’ revealed significant variation (p < 0.05).

**Table 3:** Results of AMOVA of *Anopheles arabiensis* populations inferred by microsatellite markers

Source of variation	d.f	Variance components	Percentage of variation	Fixation indices	*p value*
Among groups	2	0.06	2.75	F_CT_: 0.16	< 0.001
Among populations within groups	3	0.30	13.61	F_SC_: 0.14	< 0.001
Within population	394	1.84	83.63	0.03	0.003

Significant differentiation among populations, (F_ST_) was observed between all *An. arabiensis* pairwise comparisons for all loci (Table 4, p < 0.05). F_ST_ ranged from 0.06 to 0.24. But if Kr is excluded, and in most cases, Se as well, the comparisons will indicate moderate genetic differentiation for all. Moderate genetic differentiation was observed between *An. arabiensis* populations from My (Khartoum State) and Se (Sennar State) (F_ST_ = 0.06) and Gw (Khartoum State) and H.sh (Kassala state) (F_ST_ = 0.08). However, high genetic differentiation was observed between *An. arabiensis* populations from Kr and other populations F_ST_ = 0.17–0.24 including with H.sh which is also in the same state of Kassala. Thus, although the detailed magnitudes of population differentiation vary among population comparisons in the various analyses, in summary Kr is most distant or differentiated from other populations and followed by Se to a certain extent for several pairwise comparisons.

**Table 4:** Population differentiation of *Anopheles arabiensis* as estimated using Wright’s pairwise F_ST_

*						
Gw	*					
H.sh	0.08	*				
Hj	0.11	0.12	*			
My	0.13	0.11	0.11	*		
Se	0.18	0.16	0.17	0.06	*	
Kr	0.22	0.17	0.24	0.21	0.19	*

Gene flow (Nm) calculated from mean F_ST_ statistics ranged from 1.5 to 9.05 suggesting high gene flow between populations (Table 5) except for those involving Kr. For example, high gene flow was detected among Se (Sennar State) with My and Gw (both Khartoum State); Hj and My (both Khartoum State). Very little gene flow was observed between Kr (Kassala State) and other populations. When considering the loci outside the inversion (AGXH678, AG3H29, AG3H45 and AG3H158), F_ST_ statistics ranged from 0.026 to 0.32 with mean F_ST_ = 0.13. For loci inside inversion (AG2H603; AG2H290 and AG2H143), F_ST_ ranged from 0.019 to 0.20 with mean *F*_ST_ = 0.16. Gene flow (Nm) for loci outside the chromosomal inversions ranged from 1.04 to 43.52. The highest gene flow was between My and H.sh with Nm = 43.52, while little gene flow was found between Kr and Hj with Nm = 1.04. For loci inside the fixed chromosomal inversion, 2La, a range of Nm = 1.38 to 19.99 was observed, with the highest gene flow between Se and My with Nm = 19.99, while little gene flow was detected between Se and Kr with Nm = 1.38.

**Table 5:** Gene flow estimates between populations of *Anopheles arabiensis* in Sudan

0	Gw	H.sh	Hj	My	Se	Kr
Gw	0	426.5	5.38	15.55	323.1	443.21
H.sh	5.89	0	448.59	416.55	429.31	12.88
Hj	4.06	3.51	0	3.93	311.55	427.72
My	3.48	4.07	3.81	0	292.07	431.75
Se	2.22	2.71	2.47	9.05	0	334.01
Kr	1.69	2.30	1.50	1.88	1.97	0

Geographical distance (km) between sites

Nm, gene flow between *An. arabiensis* populations

### Population bottleneck

The Wilcoxon test (Table 6) indicated that all populations excluding Se were significant for IAM mutation-drift equilibrium (α < 0.05), but with normal L-shaped distribution. This shows that these populations had not experienced population bottleneck. However, population Hj (Khartoum State) with shifted mode suggest recent population size reduction. TPM analysis showed non-significance for all populations except Hj while the SMM analysis showed non significance for all populations.

**Table 6:** Bottleneck analysis of *An. arabiensis* populations from six areas in Sudan

Area	IAM	TPM	SMM	Mode-shift
Hj (n = 32)	0.016	0.031	0.109	Shifted
Gw (n = 33)	0.031	0.438	0.438	Normal
My (n = 34)	0.047	0.438	0.844	Normal
Se (n = 33)	0.078	0.563	0.563	Normal
H.sh (n = 34)	0.039	0.375	0.299	Normal
Kr (n = 34)	0.023	0.055	0.688	Normal

**Fig. 3:**
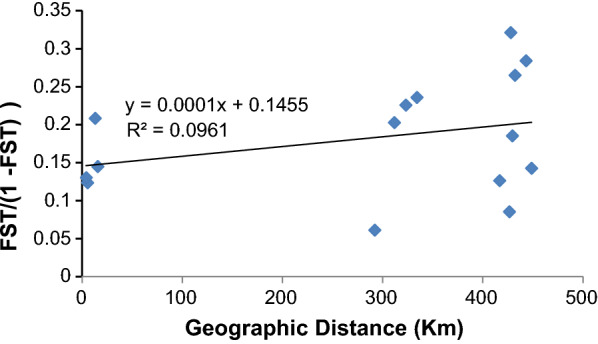
Scatter plot of the relationship between genetic and geographic distances. No significant correlation between genetic differentiation and geographic distance among *Anopheles arabiensis* populations

Although there was significant differentiation, F_ST_, between the different populations, Mantel tests showed no significant correlation (r^2^ = 0.09, p > 0.05) between genetic differentiation measured as linearized F_ST_ (F_ST_/(1 − F_ST_) and geographic distance (km) (Fig. 3). Thus, genetic and geographic distances are not correlated.

### Population structure

**Fig. 4:**
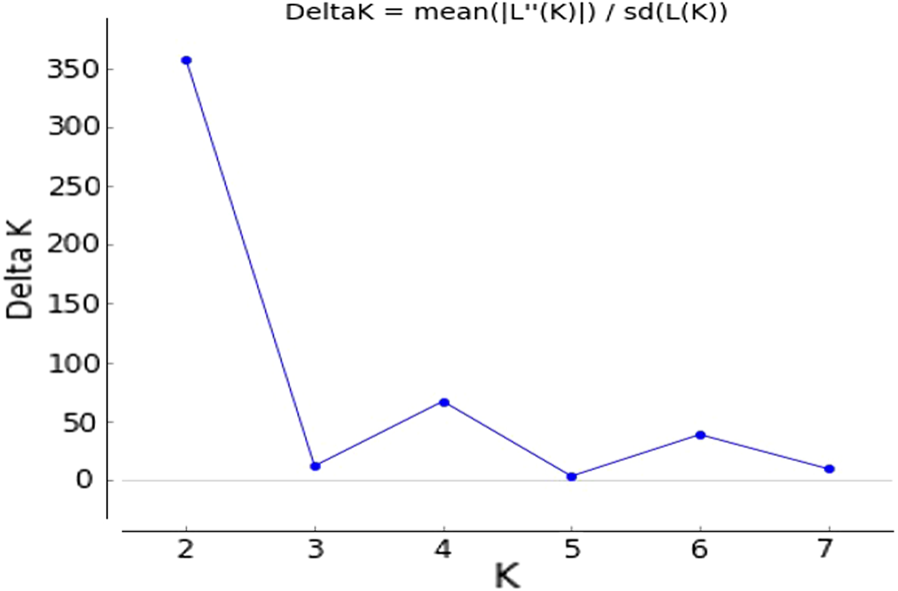
Graphical analysis using STUCTURE.HARVESTER. Representation of the data set for the populations most likely K (K = 2)

**Fig. 5:**
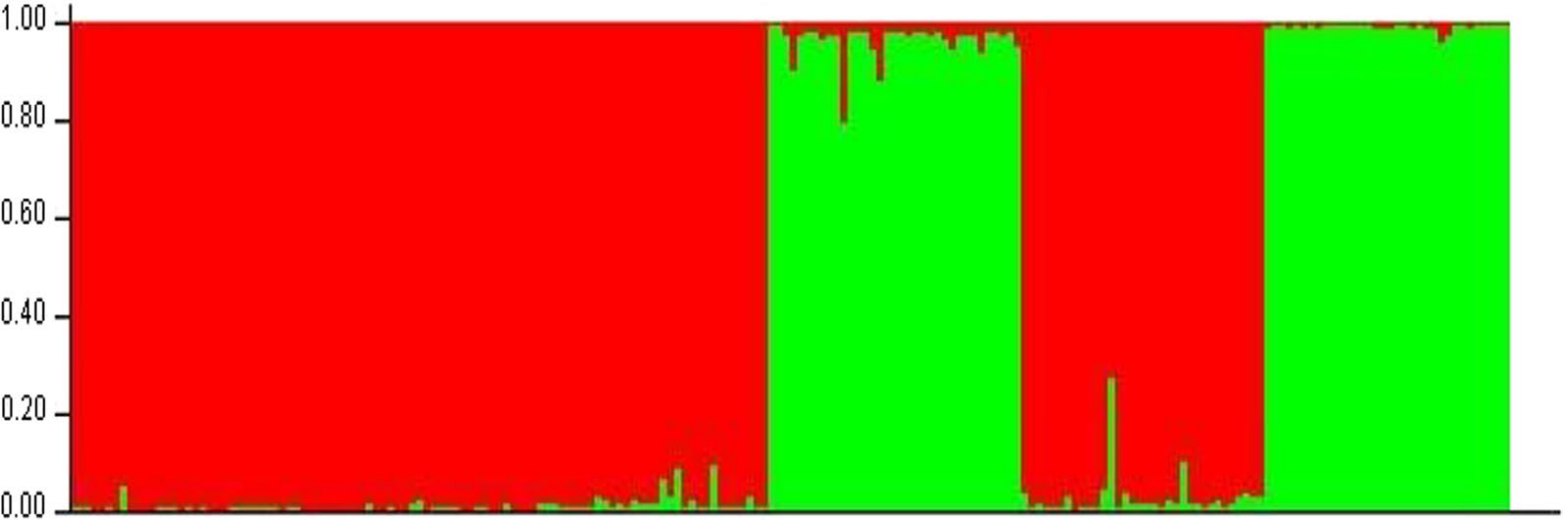
Graphical Bayesian cluster analysis using STRUCTURE. Representation of the data set for the most likely K (K = 2), where each colour corresponds to a suggested cluster. Subpopulation A (red colour) includes Hj, Gw, My and H.sh populations and subpopulation B (green colour) includes Se and Kr populations

**Fig. 6:**
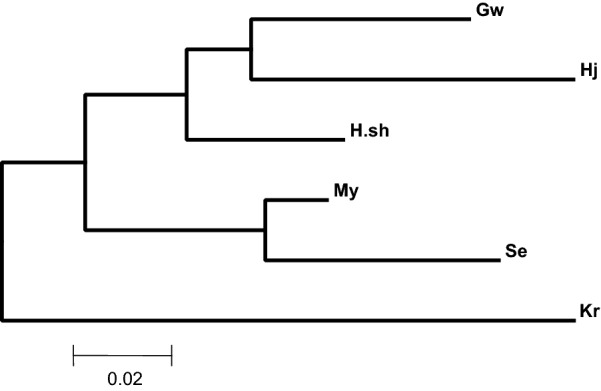
Phylogenetic tree based on Neighbour Joining method. The phylogenetic tree of *An. arabiensis* populations from Sudan. Hj, GW and H.sh, My and Se are highly genetically related, while Kr appeared to be highly differentiated from other populations

Based on the programme STRUCTURE after calculations of the delta K and plotting its value against the assumed number of populations (K = 6), a peak at *K* = 2 revealed two main clusters (Fig. 4). The dataset was further analysed by assigning individuals between the two suggested clusters. Figure 5 explains the analysis for assignment of the most likely K (*K* = 2). This analysis is in general in agreement with the F_ST_ analyses where Kr and to a lower degree, Se are distant from the other populations although My is closely related to the latter. In the F_ST_ analysis, Kr and Se were also found to be genetically distant. However, the phylogenetic tree showed that only Kr is distant from the other populations. On the other hand, *An. arabiensis* populations from Hj, GW and H.sh; My and Se are highly related genetically (Fig. 6).

## Discussion

### Allele frequency and linkage disequilibrium

In this study, a set of seven microsatellite markers, specific for *An. gambiae* [12] was used to analyse the population genetics among six *An. arabiensis* populations in Sudan. Allele number per locus ranging from 2 to 12 with an average of 7.6 alleles per locus as well as heterozygosity levels are concordant with other studies in this species conducted in Central Kenya and Madagascar [16] and Eastern Africa [25]. But higher heterozygosity values were observed in the eastern Africa Islands of Reunion and Mauritius [24, 27]. Similar values have also been obtained for other species for example in *An. gambiae* s.s. [35], *An. funestus* in east and southern Africa [53] and *Anopheles atroparvus* [54] in southern Europe. Muturi et al. [16] suggested that the level of allelic polymorphism could provide powerful measures to identify population subdivision.

Analyses of linkage disequilibrium on six *An. arabiensis* populations in Sudan suggest the existence of population subdivision. Similarly, using a different suite of microsatellite markers for *An. arabiensis* in Southern Tanzania, Ng’habi et al. [17] observed high linkage disequilibrium which they attributed to the presence of population subdivision. The significant linkage disequilibrium observed in this study could be attributed to heterozygote deficits due to several factors-departure from random mating as a result of inbreeding or selection for certain genotypes following ecological and environmental changes.

### Population differentiation and population structure

The population pairwise F_ST_ values of 0.06 to 0.24 and STRUCTURE analysis of *K* = 2, divided the six populations into two groups; comprising of Kr (Kassala State) and Se (Sennar State) in one group and H.sh, My, Hj and Gw (located in Khartoum and Kassala states) in another The F_ST_ from pooled loci reported in this study was in concordance with the reported F_ST_ values for *An. arabiensis* from Ethiopia and Eritrea [13]. Both studies detected low F_ST_ but statistically significant genetic structure in *An. arabiensis* populations. However, Donnelly and Townson [25] did not detect significant genetic structure for *An. arabiensis* populations within Malawi and Sudan. Chen et al. [55] also detected a low, but significant, genetic structure of *An. gambiae* in Lake Victoria islands (F_ST_ = 0.019) and among the six villages in the mainland (F_ST_ = 0.010). This shows that structuring pattern is influenced by numerous factors specific to the geographic locations.

In particular, a high level of genetic differentiation was found in *An. arabiensis* involving certain populations in comparison with (Kr) in the current study, in agreement with that observed between the Reunion and Mauritius Islands (F_ST_ ranged from 0.080 to 0.215), located 240 km apart in East Africa Simard et al. [27]. Similarly, high levels of genetic differentiation was detected among *An. arabiensis* populations (mean F_ST_ = 0.066) [17]. This was not in agreement with *An. gambiae* s.s. (mean F_ST_ = 0.006) which was genetically undifferentiated across the 6650 km^2^ of the Kilombero valley landscape in southern Tanzania. Kayondo et al. [56] studied genetic structure of *An. gambiae* populations among islands in north-western Lake Victoria, Uganda with F_ST_ ranging from 0.014 to 0.105 and concluded that these populations were significantly genetically differentiated [54]. Similar to F_ST_ values of 0.20 and 0.30 as described by Vicente et al. [54], Kamau et al. [57] observed F_ST_ values of 0.25 while Walton et al. [58], who studied genetic population structure and introgression in *Anopheles dirus* in South-east Asia reported F_ST_ value of 0.21–0.39. These studies highlight that pattern of population structuring may differ when comparing different sets of populations in *A. gambiae* as well as in other mosquito species.

The high differentiation of Kr from the other populations may be due to its ecology which was far from agriculture areas compared to the rest which were near or within agriculture areas. Kr population is presumably reproductively isolated due to non-random mating or ecologically isolated as an effect of the AlGash River which acts as a physical barrier separating Kr from other populations. Hemming-Schroeder et al. [59] found that cropland was an important ecological driver for gene flow of *An. arabiensis*. In agreement with this postulate, Kr which is far from any agricultural areas was instead highly differentiated from other populations. Coluzzi [60], hypothesized that inversions may play an important role in the isolation process among species in the *An. gambiae* complex and between the various forms of *An. gambiae* s.s*.* He postulated that inversions can group co-adapted gene complexes that confer adaptation in temporarily isolated peripheral populations with marginal ecological conditions. When secondary contact with the source population occurs, these inversions protect the co-adapted gene complexes from recombination, resulting in stable inversion polymorphisms and/or expansions of the population into new habitat, finally resulting in a more permanent isolation and differentiation.

High genetic similarity between My and Hj which are geographically close to each other was observed, suggesting that they may represent a single population or gene pool. According to Francois et al. [47], neighbouring populations are expected to be genetically more related than distant populations and high gene flow generally prevent local adaptation. Consequently, human transportation or wind dispersal may be the reason behind the continuous gene flow between these two localities and to a lower degree, two others namely GW and H.sh. The high gene flow found between these particular populations in this study is interesting with regards to the spread of insecticide resistance in Sudan as in consequence it also facilitated the movement of resistant mosquitoes across these connected populations. This is concordant with Kent et al. [15], who studied spatial and temporal genetic structure of *An. arabiensis* in Southern Zambia. They observed high gene flow between Macha and Namwala populations, in Southern Zambia. This is also in agreement with Muturi et al. [16], who found high gene flow among the three populations of *An. arabiensis* in Central Kenya. Failloux et al. [61] detected a significant correlation between gene flow and commercial traffic by planes and/or boats between islands on *Aedes polynesiensis* populations from islands in French Polynesia. These studies show that although the species can only fly within a limited distance, passive transportation could facilitate genetic homogeneity among distant populations.

The Mantel test showed no evidence of isolation by distance in this study, similar with that reported by Nyanjom et al. [13] in populations of *An. arabiensis* from Ethiopia and Eritrea. Isolation by distance could occur due to the limited flight range of *An. arabiensis* [62], but this was not the case for this study. It is known that the distributional range of any species is largely shaped by historical and geographical events. The species will extend its range until it reaches a physical (mountain ranges, deserts and major geographic feature) or other forms of barriers (example climatic changes). However, there are no variable ecological zones, or great physical barriers which could have led to population structuring. These results points to great impact of transportation in the genetic structure of *An. arabiensis* along the River Nile. Thus, presumably, the differentiation could largely be due to other factors than geographic distance. A similar observation was seen within *An. atroparvus* [54], where no correlation between geographic or genetic distances was detected in a study conducted in southern Europe. In agreement, Kamau et al. [21], using microsatellite loci, revealed that there were no significant relationship between geographic and genetic distances in *An. arabiensis* and *An. gambiae*, suggesting that levels of genetic differentiation are not related to geographical distance and not associated to the side on which populations were sampled in relation to the Rift Valley. Chen et al. [55] who studied population genetic structure of *An. gambiae* mosquitoes on Lake Victoria islands, west Kenya revealed a significant correlation between geographic distance and pairwise distance. On the other hand, Failloux et al. [61] found no significant effect of geographic distance on the population genetic structure on *Aedes polynesiensis* populations from islands in French Polynesia in contrast to the genetic structuring pattern of *Culex pipiens quinquefasciatus* from the same islands. In the latter species, genetic differentiation increased considerably (p < 0.01) with geographic distance [62]. These differences may be due to the variable biology of the two species as well as their histories of colonization. The current study concludes high continuous gene flow among the studied populations (except for Kr), without any signs of isolation. The high migration rate and lack of interpopulation genetic variation among the Sudanese population is attributed to the continuous human and domestic animal movement among the studied localities that facilitated the distribution of *An. arabiensis* (with the exception of Kr).

### Population size bottleneck

No severe bottleneck or reduction in population size was detected in the *An. arabiensis* populations of Sudan, except in AlHajYousif (Hj). This was evident based on the significance in Wilcoxon sign-rank test which was also supported by the “shifted mode” allele distribution. In comparison, the other populations had relatively higher rare alleles than common alleles, a sign that these populations were experiencing mutation-drift equilibrium. The situation at AlHaj Yousif (Hj) is likely due to the effective vector control programme in this area. This finding does not agree with Muturi et al. [16], who studied the population genetic structure of *An. arabiensis* in central Kenya. They did not find any evidence of genetic bottlenecks in the area under different agricultural practices. Furthermore, there was no evidence of a genetic bottleneck in *An. arabiensis* despite a drastic reduction in mosquito numbers during the drought year in southern Zambia as reported by Kent et al. [15]. This is similar to the present study, where there is a reduction in *An. arabiensis* during the dry season but no occurrence of genetic bottlenecks apart from Hj which is under vector control programmes. Hj, My and Gw in Khartoum state have very strong programmes of malarial control, the ‘Khartoum Malaria Free Initiative’ started from 2001 to 2009. The significant achievement in malarial control in Khartoum state is highly evident. For example, the percentage of malaria cases among the followers of health services decreased from 20% in 2001 to just 3.3% in 2008 and the parasitological incidence went down from 91 to just 4 per 10,000 population. Another programme was initiated in 2011 and is due to end in 2015. The objective of this initiative is to decrease malaria mortality and morbidity. Specifically, the initiative aims to reduce malaria cases by 90% by 2015 in northern Sudan compared to the number of reported cases in 2009. However, results of this study in My and Gw did not show any reduction. This could be explained due to the resistance to insecticide. Therefore, this lack of reduction in these populations suggests that they are still expanding.

Present literature on *An. arabiensis* has revealed the lack of subpopulation differentiation in relation to larval habitat utilization [22]. Lack of annual bottlenecks in response to changes in the environment has also been documented [15, 24]. Large effective population size and/or recent range expansion as opposed to group migration [24, 25] have been attributed to the lack of population structuring. This is based on several experimental studies which have reported a short flight range for this malaria vector species [26] among villages in Gambia. On the other hand, there is evidence in support of population structuring [27] from West Africa and eastern outer islands [27] of Eastern Africa. Furthermore, limited gene flow has been observed between the west and south east of the Rift Valley and in Southern Zambia [15, 21], respectively. Thus, while *An. arabiensis* displays panmixia when population numbers are high, this can be reversed when inter population geographic distances are high and habitat alterations occur, leading to genetic isolation.

Microsatellite markers analysis showed that high linkage disequilibrium detected between loci and high genetic differentiation was observed between *An. arabiensis* populations from Kr and other populations F_ST_ = 0.17–0.24. Therefore, Kr populations were more genetically isolated from the rest and from each other. Additionally, Kr may be reproductively isolated due to non-random mating or movement of people from Ethiopia to Sudan. It could also be ecologically isolated as a consequence of the Al Gash River which acts a as a physical barrier separating Kr from other populations. Therefore, Kr is easier to control compared to other populations. High gene flow was detected among Se (Sennar State) with My and Gw (both Khartoum State); Hj and My (both Khartoum State). Very little gene flow was observed between Kr (Kassala State) and other populations. All populations had not experienced population bottleneck. However, population Hj with shifted mode suggest recent population size reduction. The phylogenetic tree showed that populations Hj, GW and H.sh, My and Se are genetically closely related, while Kr appeared to be comparatively differentiated from other populations.

## Conclusion

This study concluded that genetic analysis revealed high population panmixia and continuous gene flow among the studied populations, without any signs of isolation apart from the Kr, presumably due to ecological barriers. The high migration rate and lack of interpopulation genetic variation among the Sudanese population was attributed to the continuous human and domestic animal movements among the studied localities that facilitated distribution of *An. arabiensis*. However, as discussed, both abiotic (e.g. passive transportation) and biotic (e.g. chromosome inversion) factors may influence the genetic structuring of mosquito species populations. As such, where possible it is recommended that an important consideration of any population genetics study is also to understand the underlying reasons for the pattern observed. More extensive studies on population structure and genetics of *An. arabiensis* in the other regions of Sudan using additional microsatellite loci are required to elucidate the factors that may affect gene flow for strategizing management and control of mosquito infestation in the country.

## Data Availability

All data generated or analysed during this study are included in the text.

## Abbreviations

*An*: *Anopheles*
PCR: Polymerase chain reaction
IGS: Intergenic spacer
HWE: Hardy Weinberg equilibrium
FDR: False discovery rate
IAM: Infinite allele model
TPM: Two phase models
SMM: Stepwise mutation
IRS: Indoor residual spray
ITNs: Insecticide treated bed nets
